# Dr. William B. Rice

**Published:** 1915-06

**Authors:** 


**Dr. William B. Rice,** N. Y. Homoeopathic 1863, of Lockport, died in Buffalo, May 2, aged 87. He was formerly in practice at Niagara Falls.

